# A risk-based framework to guide oversight of nucleic acid constructs

**DOI:** 10.3389/fbioe.2026.1870125

**Published:** 2026-07-15

**Authors:** Audrey Cerles, Abigail Dingus-Simmons, Rocco Casagrande

**Affiliations:** 1 Deloitte, Minneapolis, MN, United States; 2 Deloitte, Detroit, MI, United States; 3 Deloitte, Rosslyn, VA, United States

**Keywords:** biosecurity, nucleic acid synthesis screening, risk-based framework, sequences of concern, synthetic biology

## Abstract

Existing nucleic acid synthesis screening guidance refers broadly to nucleic acids and their constructs, leaving providers and policymakers uncertain which products warrant biosecurity oversight. This uncertainty can lead to gaps in protection if risky products are ungoverned, and unnecessary burdens on legitimate biological research if products that pose negligible risk are governed. This Perspective article presents a risk-informed framework for classifying synthetic nucleic acid products based on their biosecurity relevance to inform proportionate screening guidance. Drawing on input from specialists in synthetic biology and biosecurity, we developed a three-tier classification of synthetic nucleic acids, which distinguishes custom from non-custom products and groups them into high-, intermediate-, and low-risk categories. The framework suggests that the strictest scrutiny should apply to longer DNA and RNA sequences (≥50 bp/nt), including custom orders, regardless of their source, and non-custom products derived from potential pandemic viruses or other viruses regulated under the Federal Select Agent Program. Very short oligonucleotides (<30 bp/nt) and non-DNA/RNA nucleic acids should likely be excluded from routine screening. The framework also defines an intermediate-risk category for products that lie between these length thresholds or that incorporate nucleic acids into medical or other products, as these cases require further investigation and policy development. Our assessment also identifies materials not clearly covered by current guidelines as requiring oversight, such as long viral sequences in non-custom repositories. We highlight these evidence gaps and areas for investigation, and describe how this framework can help align synthesis screening oversight with the most significant biological risks.

## 1 Introduction

The regulatory landscape for nucleic acid synthesis screening is in flux. In 2024, the Office of Science and Technology Policy (OSTP) Framework for Nucleic Acid Synthesis Screening established synthesis screening as a requirement for federal funding and mandated that research institutions purchase synthetic nucleic acids only from providers who meet baseline standards (OSTP, 2024). In the first stage (planned to take effect 26 April 2025), it required providers to screen sequences 200 nucleotides (nt) or longer against Biological Select Agents and Toxins and Commerce Control List (CCL) databases and verify customer legitimacy for flagged sequences. A second stage (planned to go into effect 13 October 2026) intended to impose additional practices, including: reducing the screening window to 50 nucleotides, screening for sequences known to contribute to toxicity or pathogenicity (even when not derived from or encoding biological agents), and implementing mechanisms to detect the potential for multiple short sequences to be assembled into longer sequences of concern (SOCs).

Executive Order (EO) 14292 on Improving the Safety and Security of Biological Research, signed by President Trump on 5 May 2025, directed OSTP to revise or replace the previous OSTP framework within 90 days (The White House, 2025). It also aimed to extend screening requirements to non-federally funded research, which includes an estimated 25% of human pathogen research that is performed within the United States (US) (Greene et al., 2024). However, no new or revised guidance has been issued as of the writing of this manuscript in early 2026, leading to uncertainty around the implementation and enforcement of the 2024 OSTP framework. Multiple legislative efforts also did not move forward, including the Securing Gene Synthesis Act, Nucleic Acid Standards for Biosecurity Act, and the Gene Synthesis Safety and Security Act (Kroepke, 2025). More recently, the Biosecurity Modernization and Innovation Act of 2026 was introduced in the Senate, designating gene synthesis order and customer screening as a critical near-term stopgap, though it had not advanced as of this writing (US Congress, 2026). The international regulatory landscape is also highly fragmented, and screening rates are low: only an estimated 15% of global synthetic DNA providers currently screen sequence orders for SOCs (International Biosecurity and Biosafety Initiative for Science, 2025).

As policymakers in the US and abroad consider how to craft nucleic acid synthesis screening guidance or legislation, defining which products should fall within the scope of requiring biosecurity oversight is timely. Successive iterations of US guidance have referred broadly to nucleic acids and their constructs, without considering and categorizing the full variety of nucleic acid products and how they contribute to potential biosecurity risks. This lack of specificity may create tension between regulatory intent and practical implementation. Narrow regulation could leave gaps that enable acquisition of the materials needed to acquire or modify a pathogen. In contrast, overly inclusive regulation could impose a significant compliance burden on industry by covering routine molecular biology applications that pose minimal biosecurity risks, such as PCR primer synthesis. This burden could be crippling if implemented incorrectly; for example, Integrated DNA Technologies (IDT) alone ships approximately 90,000 custom oligos daily to more than 130,000 customers worldwide (IDT, 2023). While synthesis costs have decreased significantly, cost estimates for screening have remained fairly constant at an estimated $15 per order, representing a proportionally larger (and constantly increasing) cost burden (Sharkey et al., 2024). Overly inclusive regulation may also divert limited resources and attention from focusing on products that are associated with the most significant biological risks.

The landscape of commercially available synthetic nucleic acids encompasses several product categories. Synthetic genes (or even entire genomes) are long, double-stranded DNA constructs synthesized according to a customer-specified sequence, which are procured for applications ranging from fundamental research on protein function to industrial-scale metabolic engineering. Another significant product category is short, single-stranded oligonucleotides, which are used as key reagents in standard laboratory techniques and can also be assembled into synthetic genes. The commercial market has also expanded to include various forms of RNA, notably messenger RNA (mRNA) for the development of medical countermeasures. Finally, a range of nucleic acid analogs (e.g., locked nucleic acids) have been developed for various specialized applications.

This Perspective article presents a risk-informed framework for classifying a broad range of synthetic nucleic acid products according to their biosecurity relevance, including non-DNA/RNA nucleic acids and non-custom products. A straw version of the framework was developed based on the authors’ risk analysis and refined through discussions with eight synthetic biologists and biosecurity specialists from various backgrounds. The framework proposes criteria that policymakers can use to determine which nucleic acid products are higher risk and warrant screening requirements, which can be reasonably excluded, and which will require additional research prior to making a definitive classification.

## 2 Approach: risk-informed framework development

We developed an initial risk classification framework based on the authors’ own analysis, then refined it through structured discussions with eight specialists in nucleic acid synthesis and biosecurity, representing academic research institutions, industry, and NGO stakeholders. These discussions explored several topics, including: the relative risks posed by different nucleic acid forms (single- *versus* double-stranded DNA/RNA and non-DNA/RNA nucleic acids), appropriate length thresholds for screening requirements, treatment of products (such as engineered cell lines) that incorporate custom nucleic acids, and the distinction between custom synthesis orders and non-custom repository materials.

Among the most critical biosecurity concerns motivating synthesis screening is the potential for synthetic DNA to be misused to modify, engineer, or reconstruct viruses that are capable of global pandemic spread. To center the discussions around such a threat scenario, we considered a hypothetical malicious actor with undergraduate-level synthetic biology capabilities attempting to reconstruct a pandemic virus such as the 1918 H1N1 influenza virus strain, which has a genome approximately 13.5 kilobases long. We assumed the actor possessed basic laboratory skills, access to laboratory facilities, and the financial and other resources necessary to purchase reagents, such as oligonucleotides or longer gene fragments for assembly.

## 3 Proposed framework

Our discussions produced consensus on several core principles, which we synthesized into the tiered classification system shown in Table 1. The framework distinguishes custom from non-custom nucleic acid products and, within each category, it identifies products warranting more stringent oversight (including perhaps mandatory sequence and/or customer screening), products that require further evaluation before being definitively classified, and low-risk products that can reasonably be excluded from mandatory screening until further evidence to the contrary comes to light.

**TABLE 1:** Proposed framework of risk categorizations for nucleic acid products.

Grouping	Product	<30 bp/nt	30–49 bp/nt	≥50 bp/nt
Custom nucleic acid products	Custom ss/ds DNA and RNA (isolated or in a plasmid)	Low	Intermediate/Uncertain	High
	Custom ss/ds DNA and RNA incorporated into another product‡ or formulated for medical/consumer use§	Low	Low	Intermediate/Uncertain
Non-custom nucleic acid products	Non-custom ss/ds DNA and RNA from viruses regulated under the Federal Select Agent Program (FSAP)† and other potential pandemic viruses (isolated or in a plasmid)	Low	Intermediate/Uncertain	High
	Non-custom ss/ds DNA and RNA from FSAP† or potential pandemic viruses incorporated into another product‡ or formulated for medical/consumer use§	Low	Low	Intermediate/Uncertain
	All non-custom nucleic acids that are not from FSAP† or potential pandemic viruses	Low	Low	Low
Any source	All non-DNA/RNA analogs¶	Low	Low	Low

† This includes HHS, Overlap, and USDA Veterinary Services (VS) Select Agent viruses.

‡ This includes products where synthetic DNA is not the primary product, such as genetically modified seeds with engineered traits, cell lines containing recombinant DNA constructs, DNA barcodes embedded in luxury goods for anti-counterfeiting or supply chain tracking, gene therapy vectors, and biosensors incorporating DNA probes.

§ This includes products where nucleic acids are formulated for use in medical or other consumer applications. For example, COVID-19 mRNA vaccines contain nucleic acids encoding viral spike proteins (pathogenic determinants), and PCR diagnostic kits often contain large synthetic nucleic acids from target pathogens as positive controls.

¶ Non-DNA/RNA nucleic acids include synthetic nucleic acid analogs with modified backbones or sugar moieties that retain base-pairing capabilities. These include peptide nucleic acids (PNA), locked nucleic acids (LNA), threose nucleic acids (TNA), and morpholino oligonucleotides (PMO).

### 3.1 High-risk products

#### 3.1.1 Custom nucleic acid products

Discussion participants agreed that all single-stranded and double-stranded DNA and RNA sequences of 50 base pairs/nucleotides or longer should be subject to screening, including when these are incorporated into plasmids (whether maintained in bacterial hosts or as isolated DNA). This threshold is the same as that was set to go into effect 13 October 2026 according to the OSTP framework, and it reflects a consensus that screening sequences of this length balances technical feasibility with practical implementation concerns. While those interviewed have noted that DNA assembly from shorter oligonucleotides (∼30-mers) is technically feasible, several technical barriers with this approach are daunting, including inefficient ligation, poor yields, off-target products, and constrained overlap design. Interviewees described the assembly of longer sequences from 30-mer oligonucleotides as extremely challenging, and identified the optimal range for reliable assembly as 60–80-mers, with 50-mers emerging as a practical screening threshold.

Furthermore, screening algorithms demonstrate improved accuracy at longer lengths. The current landscape includes both proprietary and open-source tools, all of which rely on sequence comparison using a range of alignment-based algorithms against curated databases of regulated pathogens and sequences of concern, with some tools layering on functional annotation or machine-learning approaches. A recent benchmarking study of six major tools found that most currently use 50 bp/nt as their default minimum screening threshold (though several are capable of screening sequences as short as 30 bp/nt) and generally achieve a baseline performance of greater than 95% sensitivity and 97% accuracy (Laird et al., 2025). While algorithms can sometimes detect sequences of concern as short as 10 bp/nt, one interviewee noted that longer sequences (up to ∼100 bp/nt) are required to distinguish between closely related viruses, such as seasonal *versus* 1918 pandemic influenza.

In addition to technical considerations, discussion participants raised concerns about the market impact of implementing a shorter screening threshold. A 30 bp/nt cut-off would at least partially capture the vast PCR primer and sequencing market, greatly expanding both the vendor and customer base subject to screening requirements. It would also likely generate numerous false positives from routine molecular biology work.

Discussion participants also considered the challenges of implementing shorter screening thresholds. One interviewee highlighted the practical difficulty of assessing intent when a customer orders a single 30-mer oligo that matches a pathogen sequence. Such a customer could provide a reasonable and benign explanation that would be difficult to verify. To address this concern, some suggested alternative strategies, relating both to which sequences are screened and how follow-up is conducted. For instance, screening could focus on oligo pools with total lengths exceeding 1 kilobase, as these are more likely to be used in assembly projects. A tiered response system could also be implemented. For example, longer sequences (greater than ∼300 bp/nt) that match a sequence of concern could trigger more thorough follow-up measures, such as law enforcement notification, while shorter flagged sequences might be denied without follow-up. Such an approach would acknowledge that customers ordering full genes or large fragments pose a higher risk than those ordering single oligos, while ensuring the follow-up burden remains manageable for both vendors and law enforcement.

Finally, interviewees agreed DNA and RNA sequences should be subject to the same screening thresholds. Both are functionally equivalent for biosecurity purposes, as RNA can directly transfect cells or be converted to DNA via reverse transcription. One discussant noted that minor technical barriers exist, as conversion to DNA introduces error-prone steps, but these likely do not rise to the level of requiring different thresholds.

#### 3.1.2 Non-custom nucleic acid products

Non-custom nucleic acid products from sources like repositories have not traditionally been subject to screening guidance. However, discussion participants generally supported modifying guidance to encourage customer screening for DNA and RNA sequences 50 bp/nt or longer from potential pandemic pathogens and all other viruses regulated under the Federal Select Agent Program. This recommendation would broaden customer screening requirements beyond commercial providers of custom nucleic acids. For example, it would cover non-profit plasmid repositories and transfers of materials between academic laboratories. These channels could be used to transfer materials that would otherwise be subject to screening, such as genome fragments from a viral pathogen or even a complete reverse genetics system.

There was some disagreement among discussion participants about the intended scope of this category. One recommended expanding the category from potential pandemic viruses to all human-infecting viruses, arguing that genetic backbones from human pathogens could be engineered to acquire pandemic potential even if the original virus is not pandemic-capable. Another participant suggested creating a separate and additional category for genes that could reasonably increase the transmissibility or virulence of any virus.

Participants also discussed practical challenges for implementation. Academic sharing practices already involve informal screening measures, such as verifying a requestor’s identity through professional networks. Some laboratories also have procedures in place for export control approvals when sharing certain nucleic acids from listed organisms. These could both serve as models for implementation. However, the changes proposed in this framework would formalize and expand practices that are currently *ad hoc* and informal, potentially adding an administrative burden and creating conflict with established academic sharing norms. Because of this possibility, despite agreeing on the need for formalized oversight, discussion participants emphasized that the potential for unintended consequences should be closely studied, as the impacts on legitimate scientific collaboration could be substantial.

### 3.2 Low-risk products

Due to the technical challenges involved in assembling longer sequences of concern from very short oligos and the administrative burden that screening sequences of this length would impose on industry, discussion participants broadly agreed that custom and non-custom DNA and RNA sequences shorter than 30 bp/nt should be excluded from screening requirements. Interviewees also agreed synthetic nucleic acid analogs with modified backbones or sugar moieties pose a negligible biosecurity risk regardless of their length. These products include peptide nucleic acids (PNA), locked nucleic acids (LNA), threose nucleic acids (TNA), and morpholino oligonucleotides (PMO), all of which cannot practically be converted to functional DNA or RNA.

The final product category that discussion participants agreed could be excluded from screening guidance was non-custom nucleic acids that are not derived from potential pandemic pathogens or other Select Agent viruses. This applies to the vast majority of plasmids and other constructs that are stored in repositories.

### 3.3 Products requiring further evaluation

Several product types fell into an intermediate category where elicited opinion supported neither mandatory inclusion in nor exemption from screening requirements, and additional research or policy deliberation may be needed prior to making a definitive classification. These products included intermediate-length sequences and nucleic acids that are incorporated into downstream products (including medical and consumer products).

In this framework, intermediate-length sequences are single- and double-stranded DNA and RNA between 30 and 49 bp/nt. Sequences that fall within this range approach the practical lower limits for use in assembly of larger fragments while also beginning to encroach on the large PCR primer and sequencing oligonucleotide market. As previously noted, assessing the intent of a customer ordering a single flagged sequence or a small number of them is challenging for vendors. Due to the relatively high costs that are involved in follow-up screening, vendors (especially those who have not traditionally been required to screen orders) may struggle with the financial and resource burdens. Some solutions have been proposed, such as focusing on customer verification of an institutional affiliation rather than individual sequence-by-sequence review. However, since assembly of sequences of this length falls within the range of technical feasibility, future guidance should continue to consider how to balance potential biosecurity risks of these sequences with the practical feasibility of implementation and economic burden on industry. Specifically, quantitative data on assembly efficiency using oligonucleotides of varying lengths should be routinely gathered to help refine threshold recommendations.

This intermediate category also includes DNA and RNA sequences 50 bp/nt or longer that are incorporated into other products. Examples include engineered cell lines or genetically modified organisms containing recombinant DNA, anti-counterfeiting DNA barcodes in luxury goods, and artifacts from DNA information storage. Similarly, many medical applications rely on formulating nucleic acids in ways that make them difficult to misuse. For instance, COVID-19 mRNA vaccines contain SARS-CoV-2 viral spike protein sequences and PCR diagnostic kits use pathogen-derived positive controls. These products represent a feasible but indirect acquisition route for actors that are seeking to acquire a sequence. Discussion participants agreed that extracting pathogen DNA from such products would be challenging and orders-of-magnitude more costly than direct synthesis, while also requiring more extensive interaction with the product provider (which may dissuade a hostile actor who wants to remain anonymous). These factors suggest that this acquisition route is impractical for most threat scenarios. However, because these products require an initial DNA synthesis step during the manufacturing process, discussion participants recommended that screening still occur at the point of synthesis. If sequence screening finds extensive viral sequences embedded in the DNA incorporated into the product, follow-up screening could determine the rationale for including those sequences and suggest replacing them with benign sequences if there is no technical reason for their inclusion. One interviewee emphasized that regardless of the intended end use, providers should engage with any customer ordering pathogen DNA for incorporation into products.

## 4 Discussion

A revised regulatory approach to nucleic acid synthesis screening should have two main functions. It should minimize the most consequential biosecurity risks without imposing unnecessary burdens on legitimate research and the broader bioeconomy. It should also provide clear and actionable obligations for industry by reducing compliance uncertainty. To achieve these objectives, regulatory efforts should be targeted and proportionate. The tiered framework we propose is intended to be immediately practical, by clearly defining certain products as high and low risk, proportionate, by targeting biosecurity threats while avoiding unnecessary burdens on routine molecular biology applications, and adaptable over time, as the placement of products in the intermediate category is resolved through additional research, technological advancement, and policy determination.

Through discussions with practitioners in biosecurity and synthetic biology, we revealed areas of strong consensus and agreement with existing guidance documents. For example, we found broad consensus that custom DNA and RNA products 50 bp/nt or longer should be subject to screening requirements, consistent with the guidance in the 2024 OSTP framework and with the default minimum threshold used by most currently deployed screening tools (Laird et al., 2025). We also identified areas with significant gaps. For example, none of the US guidance to date has considered customer screening for purchasers of non-custom products, which represents a potential avenue for acquisition of long viral sequences. Discussion participants agreed that some non-custom products should be screened, but also that this should be limited to a small subset of sequences from potential pandemic pathogens and all other viruses regulated under the Federal Select Agent Program. There was also consensus that other product categories should be considered low risk and excluded from screening requirements, including all other non-custom products, custom sequences <30 bp/nt, and non-DNA/RNA nucleic acids. Lastly, some categories require additional research, discussion, and policy determination, including 30–49 bp/nt sequences and products that incorporate nucleic acids.

This framework is intended to represent an actionable starting point for future guidance and regulation of nucleic acid synthesis screening, balancing the level of oversight with the level of biosecurity risk, while also minimizing the burden placed on legitimate scientific research and the bioeconomy. As synthetic biology capabilities continue to evolve, especially with assistance from artificial intelligence tools, reassessment of the framework (and especially product types that fall in the intermediate category) will also be necessary.

## Data Availability

The original contributions presented in the study are included in the article/supplementary material, further inquiries can be directed to the corresponding author.
